# Management of bladder cancer recurrence following the trimodality therapy

**DOI:** 10.3389/fonc.2025.1672431

**Published:** 2025-10-01

**Authors:** Nahed Damaj, Nabih Naim, Ahmad Saad, Clarisse Kattan, Joseph Kattan

**Affiliations:** ^1^ Department of Hematology-Oncology, Hôtel Dieu de France University Hospital, Faculty of Medicine-Saint Joseph University of Beirut, Beirut, Lebanon; ^2^ Department of Medical Oncology, Institut Gustave Roussy, Villejuif, France

**Keywords:** trimodality therapy, cystectomy, chemotherapy, radiotherapy, muscle invasive bladder cancer

## Abstract

Trimodality therapy (TMT), including transurethral resection (TUR), chemotherapy (CT), and radiotherapy (RT), offers the bladder-preserving treatment option for patients with muscle-invasive bladder cancer (MIBC). TMT, once indicated, has demonstrated effective and favorable local tumor control in MIBC, with complete response rates ranging between 50% and 80%. However, residual tumor is identified on follow-up TUR in approximately 20–30% of patients, and tumor recurrence occurs in a similar proportion. In both situations, the prognosis becomes unfavorable. This manuscript reviews the current evidence regarding recurrence patterns after TMT, differentiating between non–muscle-invasive (NMIBC) and muscle-invasive (MIBC) relapses. NMIBC recurrences after TMT are often manageable with conservative treatments like repeat TURBT and intravesical BCG, without negatively impacting survival. In contrast, MIBC recurrences typically require salvage cystectomy in fit patients, offering outcomes similar to primary surgery. For those unfit for or who continue to decline cystectomy, treatment remains uncertain due to the absence of clear guidelines, and systemic therapies used in metastatic urothelial carcinoma seem commonly applied by extrapolation.

## Introduction

Bladder cancer is a relatively common malignancy, with an estimated 82,290 new cases in 2023, resulting in approximately 16,710 deaths (12,160 in men and 4,550 in women) (1). In Lebanon, bladder cancer ranks among the most prevalent cancers, accounting for 9% of all new diagnoses. It is the second most common cancer in men and the ninth in women (2). Diagnosis is typically established via transurethral resection of bladder tumor (TURBT). The predominant histologic subtype is transitional cell carcinoma (urothelial carcinoma) (3), with smoking recognized as the primary risk factor. Other contributing factors include occupational exposures, chronic indwelling catheters, recurrent bladder infections, and schistosomiasis in endemic regions.

Muscle-invasive bladder cancer (MIBC) is both prevalent and life-threatening, though it remains potentially curable with timely intervention. The prognosis is generally poor, with a 5-year overall survival (OS) rate of approximately 50%. Staging of MIBC involves abdominal and pelvic CT scans, chest imaging, and bone scans when clinically indicated. Although FDG-PET imaging can be employed to detect distant metastases, its utility in evaluating primary bladder tumors and local nodal disease is limited due to urinary excretion of FDG (4).

The primary therapeutic objective in MIBC is curative intent through a multimodal approach, which typically includes neoadjuvant chemotherapy, followed by radical cystectomy or bladder-preserving treatment (5). Bladder preservation, commonly referred to as trimodality therapy (TMT), is a viable option in carefully selected patients, particularly those unfit for or unwilling to undergo cystectomy. TMT consists of maximal TURBT followed by chemoradiotherapy (6–9). Chemoradiotherapy regimens commonly include gemcitabine, cisplatin, or 5-fluorouracil, with gemcitabine often preferred due to its favorable toxicity profile and relatively low incidence of adverse effects. Preclinical studies have shown that gemcitabine can radiosensitize a wide range of tumor cells, even at non-cytotoxic doses, thereby enhancing the effectiveness of RT (10). Radiation therapy is typically delivered with curative intent at a total dose of 64–66 Gy to the bladder over 6.5 to 7 weeks, using daily fractions of 1.8–2 Gy, 5 days per week. An alternative hypofractionated schedule could be used, such as 55 Gy in 20 fractions over 4 weeks (2.75 Gy/fraction). Elective pelvic lymph node irradiation may be included in select high-risk patients, usually to 45–50.4 Gy in 25–28 fractions (10). TMT has demonstrated favorable local tumor control, with complete response rates ranging from 50% to 80% in these MIBC patients (11).

## Materials and methods

### Search strategy

A comprehensive literature search was conducted using PubMed, Scopus, and Google Scholar databases for articles published up to May 2025. The search terms included combinations of the following keywords: *“trimodality therapy,” “TMT,” “bladder preservation,” “muscle-invasive bladder cancer,” “recurrence,” “non–muscle-invasive bladder cancer,” “NMIBC,” “salvage cystectomy,”* and *“systemic therapy.”* Boolean operators (AND, OR) were used to broaden or refine the search. Reference lists of the retrieved articles and relevant reviews were manually screened to identify additional studies.

### Eligibility criteria

Studies were eligible if:

-included patients with muscle-invasive bladder cancer treated with trimodality therapy
-reported recurrence patterns (NMIBC or MIBC) after TMT, and
-described management approaches and oncological outcomes.

Eligible study types included clinical trials, retrospective series, prospective observational studies, and meta-analyses. Case reports, editorials, conference abstracts without full data, non-English publications, and studies lacking specific recurrence outcomes were excluded.

### Data synthesis and analysis

Due to heterogeneity in study design and outcome reporting, a narrative synthesis was undertaken rather than a meta-analysis. Data were summarized qualitatively to highlight recurrence patterns after TMT, risk factors associated with recurrence, and therapeutic strategies employed. Particular attention was given to differences in prognosis and management between NMIBC and MIBC relapses, as well as outcomes of salvage cystectomy versus conservative or systemic approaches.

### Bladder outcome after TMT

Despite these promising percentages, bladder-sparing multimodal approaches are associated with a significant risk of residual or recurrent disease. Post-TMT surveillance involves urine cytology, cystoscopy, and thoracoabdominal-pelvic (TAP) CT every 3 months for the first 2 years, every 6 months up to 5 years, and annually thereafter.

### Top of form

The follow-up TURs identify residual tumors in approximately 20–30% of patients, and local recurrence occurs in a similar proportion. The prognosis is particularly poor for patients with tumors that fail to respond to initial therapy or who develop recurrent disease (11). Local recurrent disease could be non-muscle invasive bladder cancer (NMIBC) or MIBC (12–14). Tumor location, multifocality, a history of multiple transurethral resections, and a neutrophil-to-lymphocyte ratio (NLR) ≥ 2.56 were all significantly associated with an increased risk of recurrence following TMT. In contrast, patients who received early TMT after the initial diagnosis exhibited a lower recurrence rate (15).

### NMIBC recurrence approach after TMT

When recurrence is non-invasive, conservative management, similar to standard NMIBC protocols (resection with or without intravesical therapy), can be pursued. Such recurrences do not appear to impact overall prognosis or increase the risk of BCG (Bacillus Calmette-Guérin) related toxicity (16). Low-grade, noninvasive recurrences after TMT can be effectively managed with TURBT combined with intravesical therapies such as BCG or mitomycin. For NMIBC with higher risk features, such as high-grade lesions, carcinoma *in situ* (CIS), or T1 disease, treatment options include TURBT with BCG or early cystectomy, guided by clinical judgment. Of note, intravesical BCG remains effective after bladder-preserving chemoradiation, with 59% of patients with Ta grade 2/3, CIS, or T1 achieving no further recurrence. Moreover, BCG is generally well tolerated in this setting, with 68% completing induction therapy without interruption or significant toxicity (17). A study using the Princess Margaret Cancer Centre’s institutional database (University Health Network, University of Toronto), aimed to evaluate whether standard NMIBC therapies are effective for NMIBC recurrences following TMT (18). They identified 12 patients with NMIBC recurrence post-TMT between 2008 and 2019 and compared them to 60 matched controls with primary NMIBC, based on stage and grade (5:1 ratio). All TMT patients initially received weekly cisplatin (40 mg/m²), bladder-directed radiotherapy (64–66 Gy), and Lipiodol injections for localization. Recurrences were managed with conventional NMIBC treatments. Median age was higher in the TMT group (78 vs. 66 years), with median follow-up of 3.6 vs. 5.4 years. Recurrence stages in the TMT group included Ta (n=4), T1 (n=3), and CIS (n=5). The TMT group had fewer subsequent recurrences (17% vs. 63%, *P* = 0.004) and similar cystectomy rates (8% vs. 18%, *P* = 0.68). These findings suggest that NMIBC recurrence after TMT can be successfully managed using traditional intravesical and endoscopic therapies.

### MIBC recurrence approach after TMT

Salvage cystectomy is indicated for patients who are suitable surgical candidates and have failed to achieve a complete response following bladder-preserving TMT. It is particularly recommended in cases of muscle-invasive recurrence at any time post-TMT (18). Post-TMT salvage cystectomy has been shown to be a safe and viable option, with a 90-day major complication rate (Clavien-Dindo grade ≥3) of approximately 16% and a 90-day mortality rate of around 2%, rates comparable to those seen with primary radical cystectomy in patients without prior radiation. Early postoperative complications are typically cardiovascular or hematologic, while late complications often involve wound healing, uretero-enteric anastomotic strictures, or stoma-related issues. Serious complications like rectal injury and the need for colostomy are rare (3% and 1%, respectively) when the surgery is performed in experienced centers (18).

In a large series of patients undergoing cystectomy after TMT, 265 patients were analyzed specifically for outcomes following cystectomy post-TMT. 21 patients received salvage cystectomy following local relapse and the 244 received primary cystectomy post TMT. While salvage cystectomy demonstrated similar rates of intraoperative and early complications compared with primary cystectomy, it was associated with an increased risk of long-term complications. In terms of survival, no significant differences in disease-specific or overall survival were observed, supporting salvage cystectomy as an effective and safe option for appropriately selected patients who relapse after bladder-preserving therapy (19).

In another study by Rödel et al., among 42 non-responders to initial chemoradiotherapy who underwent primary cystectomy, the 5- and 10-year cancer-specific survival (CSS) rates were 21% and 18%, respectively. This was significantly worse than complete responders (41 patients) who subsequently required salvage cystectomy for MIBC recurrence, whose 5- and 10-year cancer specific survival(CSS) rates were 50% and 45%, respectively. The reasons for this difference may include more aggressive tumor biology, the adverse effects of delayed cystectomy, treatment-induced changes in tumor behavior, or selection bias, as non-responders are more likely to have unfavorable pathological features (20).

The differences in CSS between the studies may be explained by the smaller overall number of patients and the limited number who underwent primary cystectomy in the first study. If the sample size were larger, outcomes might resemble those reported in the second study, where CSS was lower for patients undergoing salvage cystectomy after relapse compared with those receiving primary cystectomy.

If a patient with recurrent MIBC after TMT remains unfit for or continues to refuse salvage cystectomy, there is currently no high-level evidence or consensus guidelines specifically supporting one treatment approach over another in this setting. However, in clinical practice, these patients are typically managed similarly to those with metastatic urothelial carcinoma (mUC). Systemic therapy options include antibody-drug conjugates (ADCs), chemotherapy, immune checkpoint inhibitors (ICIs), used either as monotherapy or in combination. The preferred initial treatment is enfortumab vedotin plus pembrolizumab if available, otherwise platinum-based chemotherapy followed by maintenance immunotherapy. For cisplatin-eligible patients, gemcitabine, cisplatin and nivolumab followed by maintenance nivolumab could be another option. Cisplatin-ineligible patients could be also treated with gemcitabine plus carboplatin followed by Avelumab or single-agent pembrolizumab. Thus, these options are usually guided by cisplatinum eligibility and the history of short free-chemotherapy interval after neoadjuvant chemotherapy.

Moreover, one can postulate that efficacy of such therapies on irradiated bladder tissue could be reduced. Radiotherapy profoundly remodels the bladder tumor microenvironment, simultaneously exerting both stimulatory and suppressive influences that can undermine subsequent systemic therapies. It induces fibrosis and vascular damage that impede drug delivery, fosters hypoxia that contributes to chemoresistance, and depletes lymphocytes, thus compromising ICIs, while also recruiting immunosuppressive cells such as regulatory T cells that blunt antitumor immunity (21, 22). This altered environment, coupled with the “tumor bed effect” where radiation-primed stroma paradoxically promotes tumor regrowth, diminishes efficacy across chemotherapy, ICIs, and ADCs.

Sapre et al. (2012) conducted a systematic review of 17 studies evaluating local recurrences in patients with MIBC who initially achieved a complete response to bladder-sparing CRT. Complete response rates ranged from 56% to 100%, while local recurrence occurred in 13–40% of cases, with NMIBC and MIBC recurrences occurring at similar frequencies. Recurrences typically developed within 18–36 months after radiotherapy, though late relapses up to 10 years were reported. NMIBC recurrences were usually managed with TURBT with or without intravesical therapy, whereas salvage cystectomy was most often advocated for MIBC recurrences. Five-year cancer-specific survival was favorable for NMIBC recurrences (50–70%) but much lower for MIBC recurrences (16–40%). Importantly, patients with recurrence had a reduced likelihood of long-term bladder preservation compared to those without recurrence. Poor prognostic factors included advanced age, incomplete resection, higher T stage, and carcinoma *in situ* at the time of radiotherapy. The authors concluded that while NMIBC recurrence following CRT can be managed conservatively with survival comparable to non-recurrent cases, MIBC recurrence carries a poor prognosis with a high likelihood of distant metastases despite salvage cystectomy, underscoring the need for cystoscopic surveillance extending up to 10 years (23). **Table 1** summarizes all the studies mentioned above.

**Table 1:** Summary of key studies on trimodality therapy (TMT) in muscle-invasive bladder cancer.

Author (Year)	Study type/sample size	TMT regimen	Follow-up duration	Recurrence type & rate	Management of recurrence	Key findings
Shipley et al., 2002 (9)	Prospective cohort, n=190	TURBT + chemoradiotherapy	Median 5 yrs	Local recurrence: NMIBC/MIBC 20–40%	NMIBC: TURBT ± intravesical therapy; MIBC: salvage cystectomy	TMT achieves bladder preservation with 50–80% complete response; salvage cystectomy effective for MIBC recurrence
Sanchez et al., 2015 (16)	Retrospective, n=66	TURBT + CRT	3–5 yrs	NMIBC recurrence 17%; MIBC recurrence 30%	NMIBC: TURBT ± BCG; MIBC: salvage cystectomy	NMIBC recurrence can be managed conservatively without affecting survival; early detection crucial
Ajib et al., 2021 (15)	Retrospective matched cohort, n=12 TMT vs 60 primary NMIBC	TURBT + weekly cisplatin + RT	Median follow-up 3.6 yrs	NMIBC recurrence 17%	TURBT + intravesical therapy	Post-TMT NMIBC recurrence shows fewer subsequent recurrences than primary NMIBC; conservative management effective
Rödel et al., 2002 (20)	Prospective, n=83	CRT ± salvage cystectomy	5–10 yrs	MIBC recurrence 50% in non-responders	Salvage cystectomy	Non-responders have worse CSS; salvage cystectomy improves outcomes in responders
Eswara et al., 2012 (17)	Retrospective, n=50	TMT then salvage cystectomy	Median follow-up 4 yrs	MIBC recurrence (all surgical)	Radical cystectomy	90-day major complication rate 16%, mortality 2%; salvage cystectomy safe and feasible
Büchser et al., 2019 (12)	Retrospective, n=124	TMT with bladder preservation	Median 60 months	Local recurrence 20–30%; NMIBC/MIBC	NMIBC: TURBT ± BCG; MIBC: cystectomy	TMT effective for local control; long-term surveillance required
Sapre et al., 2012 (25)	Systematic review, 17 studies	TMT	18–36 months (range up to 10 yrs)	NMIBC: 13–40%; MIBC: 13–40%	NMIBC: TURBT ± intravesical therapy; MIBC: salvage cystectomy	NMIBC recurrences manageable conservatively; MIBC recurrences have poorer prognosis
Alati et al., 2022 (13)	Retrospective, n=55	TMT, twice-daily hypofractionated RT	Median 36 months	Recurrence not specified	NMIBC: TURBT; MIBC: cystectomy	TMT feasible in older adults; local control acceptable
Fabiano et al., 2021 (14)	Retrospective, n=98	TMT	Median follow-up 5 yrs	Local recurrence: 25%	NMIBC: TURBT ± BCG; MIBC: salvage cystectomy	Complete response in 50–75%; salvage cystectomy outcomes comparable to primary cystectomy

Emerging evidence suggests that integrating immunotherapy with RT may enhance antitumor immunity in localized disease. The review by Zhang R et al. highlights strategies for combining immune modulation with radiotherapy, providing a framework for future clinical trials in the bladder preservation setting. While high-level evidence remains limited, these approaches may expand treatment options for patients who cannot undergo salvage cystectomy and support the development of tailored post-IO/RT management strategies (24).

## Conclusion

TMT, a combination of transurethral resection, chemotherapy, and radiotherapy, offers a bladder-preserving alternative for selected patients with MIBC, achieving complete response rates in 50–80% of cases. However, up to 30% of patients may experience local recurrences, either as NMIBC or MIBC. NMIBC recurrences following TMT can often be effectively managed with standard conservative strategies such as TURBT and intravesical therapy, mainly BCG, without negatively impacting overall outcomes. In contrast, salvage cystectomy remains the standard of care for MIBC recurrence post-TMT in surgically fit patients, with perioperative risks comparable to primary cystectomy but poorer prognosis and lower CSS. Yet, for those who are unfit for or decline cystectomy, no evidence-based consensus exists to guide management. In such cases, patients could typically treated using systemic therapies employed in metastatic urothelial carcinoma. In such cases, the use of enfortumab vedotin plus pembrolizumab, platinum-based chemotherapy with or without immunotherapy, or alternative regimens are based on cisplatin eligibility and the free-chemotherapy interval after neo-adjuvant approach. This highlights the need for further research to define optimal treatment strategies, on previously irradiated territories, for this growing subgroup of patients.
